# ZYZ-803 Mitigates Endoplasmic Reticulum Stress-Related Necroptosis after Acute Myocardial Infarction through Downregulating the RIP3-CaMKII Signaling Pathway

**DOI:** 10.1155/2019/6173685

**Published:** 2019-06-02

**Authors:** Lingling Chang, Zhijun Wang, Fenfen Ma, Bahieu Tran, Rui Zhong, Ying Xiong, Tao Dai, Jian Wu, Xiaoming Xin, Wei Guo, Ying Xie, Yicheng Mao, Yi-Zhun Zhu

**Affiliations:** ^1^Shanghai Key Laboratory of Bioactive Small Molecules, Department of Pharmacology, School of Pharmacy, Fudan University, 826 Zhangheng Road, Pudong New District, Shanghai 201203, China; ^2^State Key Laboratory of Quality Research in Chinese Medicine and School of Pharmacy, Macau University of Science and Technology, Macau; ^3^Department of Pharmacy, Pudong Hospital Affiliated to Fudan University, Shanghai, China; ^4^Institute of Biomedicine and Pharmacy, Vietnam Military Medical University, Hanoi, Vietnam; ^5^Department of Pharmacy, Huashan Hospital Affiliated to Fudan University, Shanghai, China

## Abstract

Acute myocardial infarction (AMI) is a leading cause of morbidity and mortality worldwide, and both cardiac necroptosis and endoplasmic reticulum stress (ERS) have been involved in the pathophysiology of AMI. ZYZ-803 is a hybrid molecule of a dual donor for gasotransmitters H_2_S and NO. The aim of the present study is to investigate the antinecroptosis role and potential mechanisms of ZYZ-803 in the setting of ERS during AMI injury. *In vivo*, ZYZ-803 preserves cardiac function and reduces infarct size significantly after 24-hour left coronary artery ligation through revising H_2_S and NO imbalance. In addition, ZYZ-803 relieves ERS and necroptosis in an AMI heart. *In vitro*, ZYZ-803 ameliorates ERS-related necroptosis induced by tunicamycin, and such effect has been depending on the receptor-interacting protein 3- (RIP3-) Ca^2+^-calmodulin-dependent protein kinase (CaMKII) signaling pathway. These findings have identified a novel antinecroptosis potential of ZYZ-803, providing a valuable candidate for cardioprotection in acute myocardial ischemia.

## 1. Introduction

Acute myocardial infarction (AMI) has been a leading cause of morbidity and mortality worldwide driven by an increased ageing population [1]. Damage to the coronary artery by vulnerable atherosclerotic plaques accounts for approximately 70% of all myocardial infarction events as well as by other pathogenic factors including a coronary spasm, emboli, and dissection [2–4]. The detrimental complications of AMI involving cardiac rupture, cardiogenic shock, dysrhythmic events, pericardial disease, and heart failure are responsible for the low rates of survival in patients [5]. Although pharmacological and catheter-based reperfusion (e.g., fibrinolysis and percutaneous coronary intervention) can provide mortality benefits, adverse events such as systematic bleeding and reperfusion failure of microvascular flow are still a driver of unsatisfactory outcomes. Thus, it is urgent to identify new strategies like the identification and characterization of novel cardioprotective drug candidates that can be developed for AMI intervention.

Pathologically, vast cardiac cell death following sharp ischemic insult is a hallmark process of AMI [5]. Programmed cell death and passive occurring necrosis are traditionally thought as the two main types of ischemic cardiomyocyte death, and the former has become a key target for its gene-directed and regulated property in the field of cardioprotective study. Notably, necroptosis, or regulated necrosis, has recently been shown to be an important factor linked to ischemic heart injury [6–11]. As such, necroptosis holds particular appeal as a pharmacological target for cardioprotection. Important in necroptotic signaling is receptor-interaction protein 3 (RIP3). This protein serves as the core player in cardiac necroptosis along with RIP1-RIP3-MLKL (mixed lineage kinase domain-like protein) and RIP3-CaMKII- (Ca^2+^-calmodulin-dependent protein kinase-) dependent pathway [9, 11, 12]. Targeting to these signaling pathways offers myocardial protection [8, 10, 13–17]. As such, targeting of these signaling systems could be important in the treatment of AMI.

Cardiomyocytes are rich in endoplasmic reticulum (ER) needed for contractive processes and metabolism. For this reason, cardiomyocytes are susceptible to endoplasmic reticulum stress (ERS) induced by exogenous injury such as ischemia and hypoxia. ERS is an evolutionarily conserved response to functional disturbances in cell ER function induced by genetic and environmental insults that can lead to cell death. The hallmark of ERS could be indicated by upregulation of some related genes such as activating transcription factor 6 (ATF6), CCAAT/enhancer-binding protein homologous protein (CHOP), glucose-related protein 78 (GRP78), and calreticulin [18]. A significant player in these processes is changes in cellular calcium pools; particularly, overload of intracellular Ca^2+^ is a hallmark of aberrant ERS [19]. Numerous researchers have revealed the pathophysiologic role of ERS in various diseases such as diabetes, neurodegeneration, cancer, and ischemic heart disease [20, 21]. In myocardial infarction (MI), metabolic disturbance resulted from acute hypoxia, and hypoglycemia frequently generates excessive misfolded protein accumulation, leading to aberrant ERS. This process can initiate secondary cell death, usually via the induction of apoptosis [21–25]. Although it has been reported that necroptosis is an alternative modality of ERS-induced downstream cell death in the L929 cell line [26], it remains unverified in myocardial cells. Furthermore, inhibition of ERS can improve insulted cardiac function, reduce adverse remodeling, and decrease cardiac apoptosis during MI [27, 28]. So, targeting to ERS and its possible downstream necroptosis should be a valuable way for MI prevention.

ZYZ-803 is a hybrid molecule synthesized by our laboratory that consists of S-propargyl-cysteine (a H_2_S donor) and furoxan (a NO donor). This molecule is designed to slowly release the gasotransmitters of H_2_S and NO that are important in cardiovascular function [29, 30]. It has been demonstrated that ZYZ-803 has a vasorelaxant effect on rat aortic rings [29] and angiogenesis role in the mouse ischemic hind limb model [31]. ZYZ-803 also showed cardioprotective potential in isoprenaline-induced heart failure [32]. The effects of ZYZ-803 are mediated via the interplay between H_2_S and NO that act cooperatively to prevent cardiac damage. Since dysregulation of H_2_S and NO homeostases exists during myocardial infarction, and aberrant ERS should possibly result in downstream cardiac necroptosis, targeting to ERS-related myocardial necroptosis during AMI as well as H_2_S and NO imbalance is a promising cardioprotective scheme. In the current study, we intend to investigate the cardioprotective effects of ZYZ-803 on acute myocardial infarction both *in vivo* and *in vitro*. Additionally, the underlying mechanisms of action ascribed to ZYZ-803 have been addressed with a focus on ERS-related myocardial necroptosis and the subsequent signaling pathway involved.

## 2. Materials and Methods

### 2.1. Animals and Experimental Protocols

Adult male C57BL/6 mice (8 weeks) were acquired from the Sippr-BK Experimental Animal Center (Shanghai, China) and housed under pathogen-free conditions with free access to food and water. All animal procedures were performed according to the Guide for the Care and Use of Laboratory Animals published by the National Institutes of Health and approved by the Ethics Committee of Experimental Research, Shanghai Medical College, Fudan University. Mice were randomly assigned into 5 groups: sham group, model group, and (2, 4, and 8 mg/kg/day) ZYZ-803-pretreated group. ZYZ-803 was dissolved in 0.1% DMSO for intraperitoneal administration for 5 days before myocardial infarction operation. The model and ZYZ-803-pretreated groups had induced myocardial infarction by permanent ligation of the left anterior descending coronary artery following previous description [33]. Briefly, mice were intubated by a 22-gauge intravenous catheter and ventilated with a mouse ventilator after isoflurane anesthetization. The epigastric hair of the mouse was sheared to expose the surgical field, and then, the thoracic cavity was opened at the third intercostal space in order to permanently ligate the left anterior descending coronary artery using the 8-0 Prolene suture at 1-2 mm distal below the left auricular appendix. When the top of ventriculus sinister was observed blanching, it was confirmed infarction. Animals in the sham group were just performed with thoracotomy without ligation to the left anterior descending coronary artery.

### 2.2. Echocardiography

After 24-hour infarction, mice were anesthetized using 1.5% isoflurane in a 95% mixed oxygen and fastened on a heating pad. Parameters for heart function were acquired by transthoracic two-dimensionally directed M-mode echocardiography dynamically using a high-resolution ultrasound system (Vevo 770; VisualSonics Inc., Toronto, ON, Canada) with a mechanical scan probe, and the transducers for the ventricular structure were at frequency of 10 MHz to provide spatial resolutions. Left ventricular ejection fraction (LVEF, %), left ventricular fractional shortening (LVFS, %), left ventricular systolic diameter (LVSD, mm), and left ventricular systolic volume (LVSV, *μ*l) were calculated automatically by the system.

### 2.3. Infarct Size

Infarct size was determined by TTC and Evans blue dye. In brief, after 24-hour infarction, mice were anesthetized by 1.5% isoflurane, intubated using a catheter, and ventilated with a mouse ventilator. Thoracotomy was done again, and 2% Evans blue in PBS (pH 7.4) was slowly perfused into ventriculus sinister from the tip of hearts to visualize the area at risk (AAR) until the mouth and limbs of mice turned into blue. Then, mouse hearts were harvested rapidly and excised into 6 sections before they were incubated in 1% TTC solution (in PBS, pH 7.4) for 15 minutes at 37°C. And then, heart sections were fixed in 4% paraformaldehyde for 24 hours until they were digitally scanned by a scanistor. Scanned photographs were assessed by ImageJ software (NIH, Boston, USA). Infarct size (IS) was calculated as the ratio of IS/AAR.

### 2.4. Histopathology

After 72-hour AMI, the mouse hearts were isolated and fixed in 4% paraformaldehyde (pH 7.4) for 24 hours at room temperature and then embedded in paraffin in order to be serially divided into sections of 5 *μ*m thickness. Standard hematoxylin and eosin (HE) staining was performed to observe histopathological changes, and Sirius red dye was applied to evaluate collagen deposition. The protocols for HE staining and Sirius red dye followed the manufacturers' direction of the corresponding kits (G1120 and G1470, Beijing Solarbio Science & Technology Co. Ltd., China), and then, sections were photographed by an optical microscope (Zeiss Inc., Oberkochen, Germany).

### 2.5. Measurements of H_2_S and NO Concentrations in Serum and Heart Tissues

H_2_S concentration in serum was detected according to the previous method [32]. In brief, 75 *μ*l serum was mixed in 250 *μ*l zinc acetate (1%, *w*/*v*), 425 *μ*l deionized water, 133 *μ*l N-dimethyl-p-phenylenediamine sulfate (20 mmol/l in 7.2 mmol/l HCl), and 133 *μ*l FeCl_3_ (30 mmol/l in 1.2 mmol/l HCl), and then, the mixture was incubated for 10 minutes at room temperature. Next, 250 *μ*l trichloroacetic acid (10%) was added and then centrifuged at 14,000 rpm for 5 minutes. The absorbance at 670 nm was set to detect the changes in each sample. The H_2_S level in mouse heart tissues was evaluated by a commercial ELISA (enzyme-linked immunosorbent assay) kit (N06478, Shanghai Jining Company, China) for mouse H_2_S according to the manufacturer's direction. The concentration of NO in serum and heart tissues was assayed by the commercial Total Nitric Oxide Assay Kit (S0023, Beyotime Institute of Biotechnology, China) in accordance with the manufacturer's protocol.

### 2.6. Primary Neonatal Rat Ventricular Cardiomyocyte

Primary neonatal rat ventricular cardiomyocytes (NRVCs) were isolated from 1-day neonatal rats using the Pierce Primary Cardiomyocyte Isolation Kit (88281, Pierce Biotechnology, Thermo Scientific, USA) according to the manufacturer's instructions. Briefly, the freshly dissected hearts from newborn rats were placed in cold Hank's Balanced Salt Solution (HBSS) and minced into 1-3 mm^3^ pieces. Then, the minced heart tissues were washed in cold HBSS twice to remove blood before adding cardiomyocyte isolation enzyme mixture (including papain and thermolysin). The tissues with enzyme mixture solution were incubated in a 37°C incubator for 30 minutes. After gently removing the enzyme solution, the tissues were washed twice in cold HBSS. The complete Dulbecco's modified Eagle medium (DMEM) for primary cardiomyocyte isolation was added to break the tissue by pipetting up and down 25-30 times until the tissues were primarily a single-cell suspension. The appropriate cell suspension was plated with 0.1% cardiomyocyte growth supplement into culture vessels and incubated in a 5% CO_2_ incubator at 37°C until next treatment procedures.

### 2.7. Cell Viability Assay

Cell viability after ZYZ-803 and/or tunicamycin administration was assayed by MTT (3-(4,5-dimethylthiazol-2-yl)-2,5-diphenyltetrazolium bromide) as previously described [31]. Primary cardiomyocytes were cultured in 96-well plates and incubated for 12 hours. After pretreatment with designed concentration of ZYZ-803 for 1 hour and following 48-hour tunicamycin (Tuni) administration, 0.5 mg/ml MTT was added into cell DMEM for another 4-hour incubation. Then, dimethylsulfoxide was added into wells to dissolve the formazan crystal. Absorbance at 570 nm in a microplate reader (M1000, TECAN, Austria GmbH, Austria) was set to measure changes in each group.

### 2.8. LDH and cTnI Measurement

Serum LDH and cTnI were assayed using related commercial assay kits (C0016, Beyotime Institute of Biotechnology, China, and E-EL-M1203c, Elabscience Biotechnology Co. Ltd., China, respectively) according to the manufacturers' instructions. For LDH detection, the cell supernatant or serum was incubated with prepared LDH working solution in the dark for 30 minutes at room temperature. Absorbance at 490 nm was measured using a microplate reader (M1000, TECAN, Austria GmbH, Austria). For the cTnI assay, serum was added into the well plate precoated with the mouse cTnI specific antibody; then, a biotinylated antibody specific for cTnI and avidin-horseradish peroxidase (HRP) conjugate were added successively to each well plate and incubated. After free components were washed away, the substrate solution was added into each well. Finally, stop solution was added to terminate the enzyme-substrate reaction, and then, the color turns yellow. The optical density was measured spectrophotometrically at 450 nm using a microplate reader (M1000, TECAN, Austria GmbH, Austria).

### 2.9. Hoechst 33342 and Propidium Iodide Staining

Cellular necrosis was evaluated using the Apoptosis and Necrosis Assay Kit (C1056, Beyotime Institute of Biotechnology, China) according to the manufacturer's direction. Briefly, cardiomyocytes were cultured on slides for 72 hours. After designed administration of ZYZ-803 and/or Tuni, cells were washed twice in PBS and added with Hoechst and propidium iodide (PI). Then, cell slides were incubated on ice for 20-30 minutes and photographed using a fluorescent microscope (Zeiss Inc., Oberkochen, Germany). Cells with strong red and blue fluorescence were defined as necrosis positive. ImageJ software (NIH, Bethesda, MD, USA) was used to count positive necrotic cells.

### 2.10. Intracellular [Ca^2+^]_i_ Evaluation

Overload of intracellular Ca^2+^ concentration is defined as a marker of ERS and cell injury [29]. Fluo-3/AM (Dojindo Lab, USA) is a fluorescent Ca^2+^-indicator probe and was used to measure the level of intracellular [Ca^2+^]_i_. After designed treatments with ZYZ-803 and/or Tuni, cardiomyocytes were washed with cold HBSS and loaded with 5 *μ*Μ fluo-3/AM for 30 min at 37°C. Then, cells were washed twice to remove the dye before another 30 minutes was allowed to hydrolyze fluo-3/AM. A confocal microscope (Zeiss Inc., Oberkochen, Germany) at a wavelength of 480 nm excitation/525 nm emission was used to photograph the intracellular [Ca^2+^]_i_. Fluorescence intensity was analyzed by ImageJ software (NIH, Bethesda, MD, USA).

### 2.11. Small RNA Interference

Small interfering (si)RNA oligos for rat RIP3 gene were purchased from Thermo Fisher (Silencer® Select siRNA s80756). Target sequence for RIP3 is 5′CGUGAACUCGAAGAAGAUATT3′. For RNA interference, cardiomyocytes in six-well plates were transfected with final concentration of 10 nM siRNA using the X-tremeGENE siRNA Transfection Reagent (Cat. No. 04476093001, Roche) according to the manufacturers' instructions. The medium was replaced at 6-hour posttransfection, and silencing efficiency was determined by real-time PCR and Western blot 48 hours after transfection.

### 2.12. Real-Time Quantitative RT-PCR

Cardiomyocyte total RNA was abstracted using TRIzol (Invitrogen, Carlsbad, CA, USA) in accordance with the manufacturer's direction. Reverse transcription cDNA was prepared using the Primer Script RT Reagent Kit (TaKaRa Biotechnology Co. Ltd., Dalian, China). An amplified outcome of PCR was detected using the CFX96 Real-Time PCR Detection System (Bio-Rad). Primer sequences of RIP3 are forward primer: AAACCACTGAGCGAGCATCC and reverse primer: TCCCTGAAATGTGGACAGGC.

### 2.13. Coimmunoprecipitation

Immunoprecipitation (IP) lysis buffer (20 mM Tris-HCl, 150 mM NaCl, and 1% Triton X-100, pH 7.5) was used to lyse mouse heart tissues, and the lysis was then centrifuged at 12,000 rpm for 10 minutes to remove cell debris. Supernatant of lysis was incubated with IgG antibody or target protein antibody and Protein A/G Plus-Agarose (Santa Cruz Biotechnology) at 4°C on a shaker overnight. The immunoprecipitated complex was collected by centrifugation and washed 4 times. The final pellet was resuspended in sample buffer for the following SDS-PAGE analysis.

### 2.14. Protein Immunoblotting

Total proteins of cells were extracted by the NuPAGE 1x LDS Sample Buffer (Invitrogen, USA), while tissue proteins were extracted in RIPA buffer (1% Triton X-100, 150 mM NaCl, 5 mM EDTA, and 10 mM Tris-HCl, pH 7.0) containing phosphatase and protease inhibitor cocktail (Thermo Scientific, USA). The protein extracts were boiled in 96°C water bath before loading onto sodium dodecyl sulfate polyacrylamide gels and transferring to nitrocellulose membranes (PALL, USA). Then, the transferred membranes were blocked in 5% nonfat milk in TBS (Amresco, Solon, OH, USA) containing 0.1% Tween-20 for 2 hours at room temperature. Next, the membranes were incubated with the primary antibodies of target proteins overnight at 4°C, followed by binding with HRP-conjugated or fluorescent secondary antibodies. Final detection of protein blots was performed using the Immobilon Western Chemiluminescent HRP Substrate (Millipore, Billerica, MA, USA) or Odyssey imaging system (LI-COR Biosciences, USA).

### 2.15. Cell Immunofluorescent

NRVCs were cultured in confocal cell dishes for 48 hours before ZYZ-803 pretreatment and Tuni-induced injury. Then, cell dishes were washed in PBS twice and fixed in 4% paraformaldehyde for 20 minutes at room temperature. Next, after being washed in PBS, 0.2% Triton X-100 was used to permeate cells for 5 minutes. Cells were blocked in 5% BSA for 30 minutes at room temperature before successive incubation with primary antibodies and fluorescent secondary antibodies. Finally, cell dishes were covered with fluorescent quencher and observed by a confocal laser scanning microscope (Carl Zeiss, USA).

### 2.16. Drugs and Reagents

ZYZ-803 was synthesized and purified as described previously [29]. Tunicamycin (HYA0098) was purchased from MedChem Express (Shanghai, China). TTC and Evans blue were purchased from Sigma-Aldrich (St. Louis, MO, USA). Antibodies to CSE and RIP3 were purchased from Santa Cruz Biotechnology (Santa Cruz, CA, USA). Antibodies to calreticulin, ATF6, CHOP, phosphorylated CaMKII, and total-CaMKII were purchased from Cell Signaling Technology (Beverly, MA, USA). Antibody to MLKL was purchased from Signalway Antibody (College Park, MD, USA). Antibodies to GRP78, RIP1, and CBS were purchased from Proteintech (Rosemont, IL, USA). Antibody to GAPDH (MB001) was purchased from Bioworld (Nanjing, China). HRP-conjugated secondary antibodies were from Jackson Laboratories (West Grove, PA, USA). Goat anti-rabbit and donkey anti-mouse fluorescent secondary antibodies were purchased from LI-COR (Lincoln, NE, USA).

### 2.17. Statistical Analysis

The software GraphPad Prism v.5.01 (GraphPad Software, La Jolla, CA, USA) was used to analyze experimental data. Values are expressed as mean ± SEM. Comparison between two groups was performed using the Student *t*-test (two-tailed), while variance among multiple groups was assessed by one-way ANOVA with Bonferroni *post hoc* analysis. Statistical significance for each test was defined as ^∗^ *P* < 0.05 at least.

## 3. Results

### 3.1. ZYZ-803 Dose-Dependently Blocks Deterioration of the Cardiac Function after AMI

**Figure 1:**
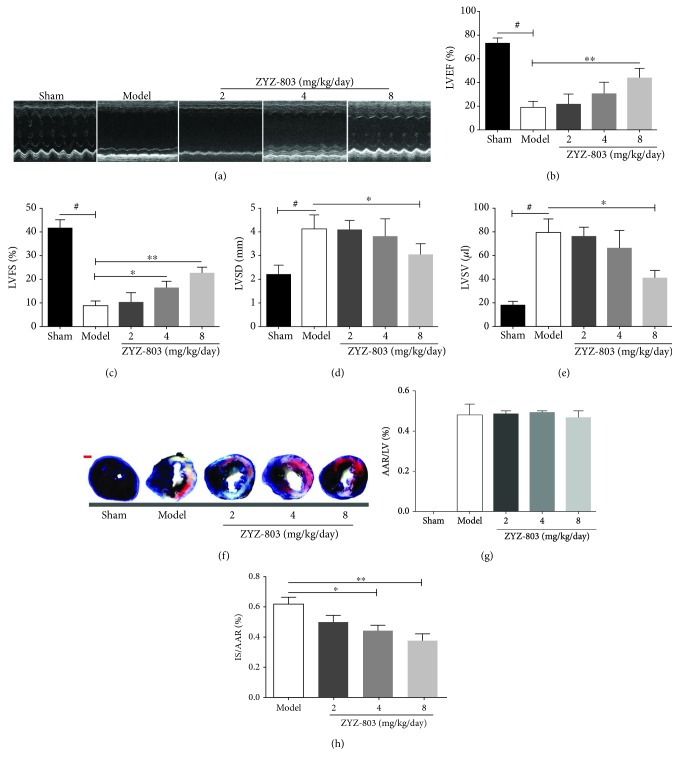
Pretreatment of ZYZ-803 preserves the left ventricular function and palliates the myocardial infarction scale. Mice were suffered from 24-hour ligation of the left anterior descending coronary artery after 5-day ZYZ-803 preconditioning. (a) Representative echocardiographs from transthoracic two-dimensionally directed M-mode echocardiography. (b–e) Statistical data of (b) left ventricular ejection fraction (LVEF (%)), (c) left ventricular fractional shortening (LVFS (%)), (d) left ventricular systolic diameter (LVSD (mm)), and (e) left ventricular systolic volume (LVSV (*μ*l)), assessed by echocardiography (*n* = 6 mice in each group). Data are mean ± SEM, one-way ANOVA. ^#^ *P* < 0.001 versus sham group; ^∗∗^ *P* < 0.01 and ^∗^ *P* < 0.05 compared with the model group. (f) Representative photographs for IS and AAR in the heart by TTC and Evans blue dye from each group. Scale bar (red): 1 mm. Quantitative data of (g) AAR/LV and (h) IS/AAR. AAR: area at risk; IS: infarct size; LV: left ventricle (*n* = 6 mice per group). Compared to the model group, ^∗∗^ *P* < 0.01 and ^∗^ *P* < 0.05. Data are mean ± SEM, one-way ANOVA.

In order to investigate the protective potential of ZYZ-803 against AMI, based on our previous study [31] and preexperiments (Figure S1), we chose 2, 4, and 8 mg/kg/day ZYZ-803 to pretreat mice daily and intraperitoneally for 5 days before permanent ligation of the left anterior descending coronary artery. Cardiac function of experimental mice indicated by regional ventricular wall motion was assessed by transthoracic echocardiography. As displayed from the echocardiographic results, deterioration of the mouse left ventricle systolic function could be obviously observed in the model group compared to the sham one, which was rescued by ZYZ-803 in a dose-dependent manner (Figure 1(a)). Similarly, the statistical values of cardiac parameters LVEF, LVFS, LVSD, and LVSV were reduced in groups of ZYZ-803 pretreatment when compared to the model group (Figures 1(b)–1(e) and Table S1); especially, these parameters in the high-dosage group (8 mg/kg/day) were much more significantly decreased (^∗^ *P* < 0.05 and ^∗∗^ *P* < 0.01 compared with the model). In addition, ZYZ-803 pretreatment showed no side effect to cardiac function in sham mice (Table S1), and the heart rate of mice in each group after AMI and ZYZ-803 pretreatment presented no apparent change (Figure S4). These data indicated that ZYZ-803 could play a preservation role in the injured cardiac function after AMI.

According to clinical research, myocardial infarct size is a detrimental factor for AMI to develop into heart failure [34]. So, it is quite necessary to evaluate the impact of ZYZ-803 on infarction scale change in order to further confirm its protective potential after AMI. TTC-Evans blue dye has usually been used to measure the infarction scale by presenting infarct size (IS) with white color and area at risk (RRA) without blue color. As the results show, there was no significant difference between the model and ZYZ-803 groups in RRA (Figures 1(f) and 1(g)). However, ZYZ-803 markedly decreased the IS/AAR ratio at the dosages of 4 and 8 mg/kg/day as compared to the model group (Figures 1(f) and 1(h); ^∗^ *P* < 0.05, ^∗∗^ *P* < 0.01), implicating ZYZ-803 improved cardiac function after AMI.

### 3.2. ZYZ-803 Decreases the Level of Serum Biomarkers for Myocardial Necrosis and Improves the Compromised Cardiac Compliance

**Figure 2:**
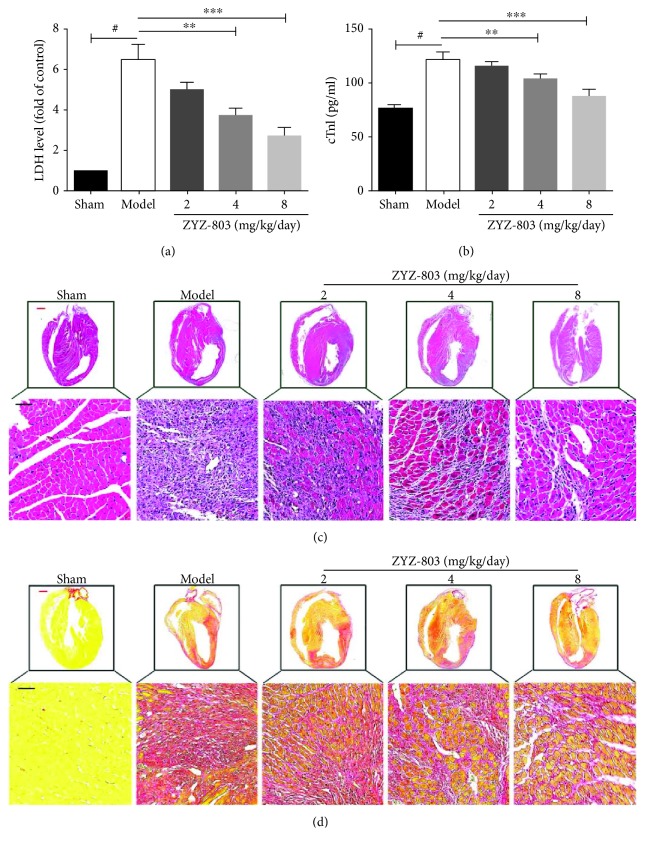
ZYZ-803 decreases the level of serum biomarkers for myocardial necrosis and improves the compromised cardiac compliance. (a, b) Both serum LDH and cTnI were decreased by ZYZ-803, *n* = 6 mice per group; compared to the model group, ^∗∗^ *P* < 0.01 and ^∗∗∗^ *P* < 0.001. Data are mean ± SEM, one-way ANOVA. (c) Hematoxylin and eosin staining of heart tissue sections from experimental animals to evaluate the severity of histopathologic changes after AMI. (d) Sirius red dye for evaluation of collagen deposition after mouse AMI. Red bars: 2.5 mm; black bars: 100 *μ*m.

In the clinical practice, evaluation of serum biomarkers is a cornerstone for AMI diagnosis [35, 36]. LDH has been a broad-spectrum enzymatic marker for myocardial injury, and cTnI, a component of the myocardial cell, is a specifically sensitive marker for myocardial necrosis. So, we chose these two biomarkers for further evaluation. From the data in Figures 2(a) and 2(b), apparently, the serum LDH and cTnI levels in ZYZ-803 groups were decreased compared to the model group (^∗∗^ *P* < 0.05, ^∗∗∗^ *P* < 0.01), implying that ZYZ-803 protected against myocardial necrosis.

At the necropsy level, histopathologic change of heart tissue has been considered as a key indicator of AMI injury in animal experiments. Because histopathological change results from a subacute pathological process during AMI [5], mouse hearts were harvested after 72-hour AMI, and then, HE staining and Sirius red dye were utilized to testify the effect of ZYZ-803 on heart histopathology. As shown in Figures 2(c) and 2(d), when compared to the sham group, there were significant catastrophic changes of disorganized cell arrangement, neutrophil infiltration and cytolysis from HE staining, and plenty of collagen deposition indicating myocardial fibrosis from Sirius red dye in the heart tissues of the model group. However, ZYZ-803 pretreatment could positively improve these compromised histopathological changes of cardiac compliance in comparison with the model one (Figures 2(c) and 2(d)). These results further demonstrated the protective potential of ZYZ-803 in AMI.

### 3.3. Protection of ZYZ-803 against AMI Injury Is Mediated by Its Counterbalancing to CBS/CSE/eNOS Synthase Systems

**Figure 3:**
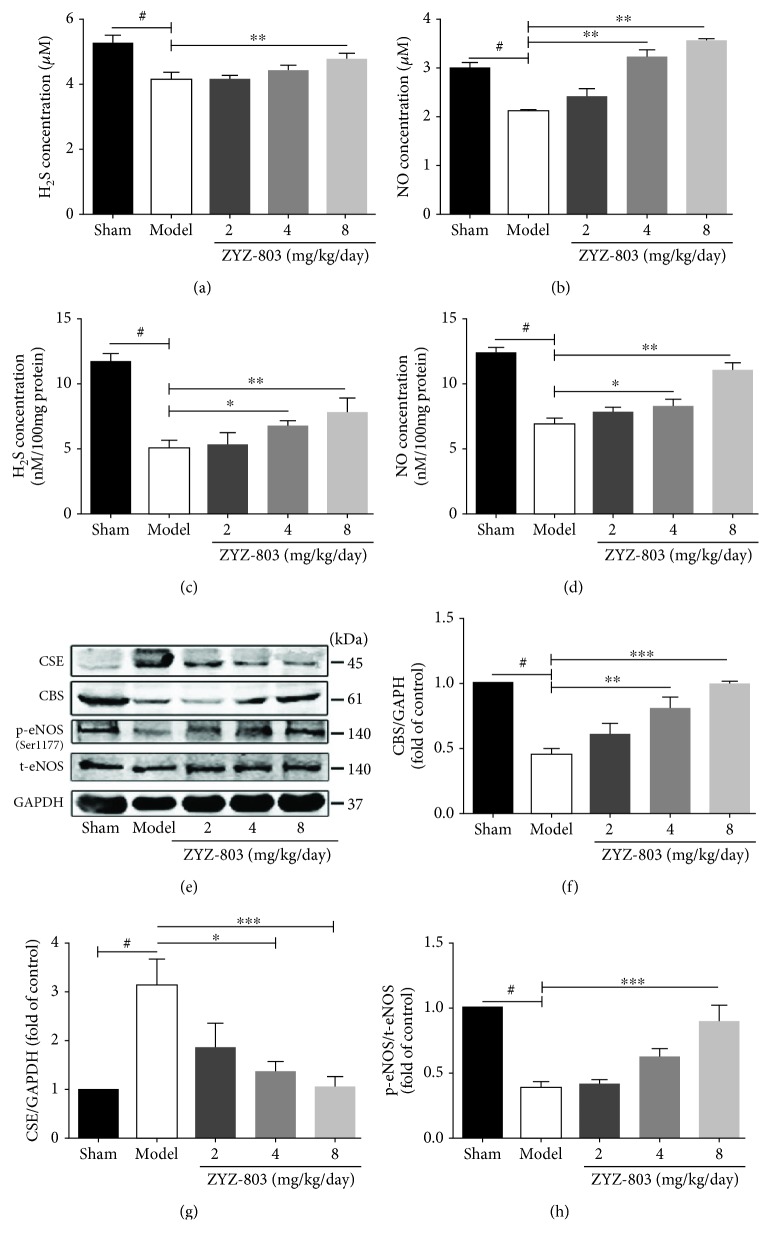
ZYZ-803 preserves H_2_S and NO homeostases through regulating balance of the endogenous enzyme levels. (a, b) Averaged data of serum H_2_S and NO concentration in experimental mice (*n* = 6 mice each group). (c, d) Averaged data of H_2_S and NO contents in mouse heart tissues (*n* = 6 mice each group). (c) Representative graphs of protein expression detected by Western blotting. (d–f) Statistical analysis graphs of p-eNOS/t-eNOS, CBS, and CSE; each protein is normalized by GAPDH (*n* = 4 independent experiments). Data are presented as mean ± SEM, one-way ANOVA. ^#^ *P* < 0.01 versus sham; compared to the model group, ^∗^ *P* < 0.05, ^∗∗^ *P* < 0.01, and ^∗∗∗^ *P* < 0.001.

The regulation of ZYZ-803 to H_2_S and NO has been demonstrated in the mouse ischemic hind limb and heart failure [31, 32]. However, whether ZYZ-803 could protect against AMI injury through the regulation to H_2_S and NO systems has not been confirmed. So here, the concentration of H_2_S and NO in mouse serum and heart tissues was assessed, and the expression and activity of related synthases were detected. Figures 3(a) and 3(b) showed the results of serum H_2_S and NO levels by defined chemical methods, and disastrously decreased levels of serum H_2_S and NO in AMI mice were observed in the model group, which were both apparently reversed by ZYZ-803 pretreatment (^#^ *P* < 0.01, ^∗∗^ *P* < 0.01). Similarly, decreased H_2_S and NO concentrations were blocked by ZYZ-803 pretreatment in mouse heart tissues (Figures 3(c) and 3(d); ^∗^ *P* < 0.05, ^∗∗^ *P* < 0.01). As to the change of related synthases, abnormal expressions of H_2_S synthase cystathionine-*γ*-lyase (CSE) and cystathionine *β*-synthase (CBS) were found in the model mice, and similar results were verified in the activity of endothelial NO synthase (eNOS), the main synthase of NO in the heart (Figures 3(e)–3(h); ^#^ *P* < 0.01). However, these enzymatic changes were expectedly salvaged by preadministration of ZYZ-803 as demonstrated in Figures 3(e)–3(h) (^∗^ *P* < 0.05, ^∗∗^ *P* < 0.01, and ^∗∗∗^ *P* < 0.001). These results reflected that the dysregulation of H_2_S and NO homeostases during AMI and ZYZ-803 pretreatment could correct this imbalanced H_2_S-NO systems to protect against myocardial injury.

### 3.4. Cardiac ERS Injury and Necroptosis during AMI Are Mitigated by ZYZ-803

**Figure 4:**
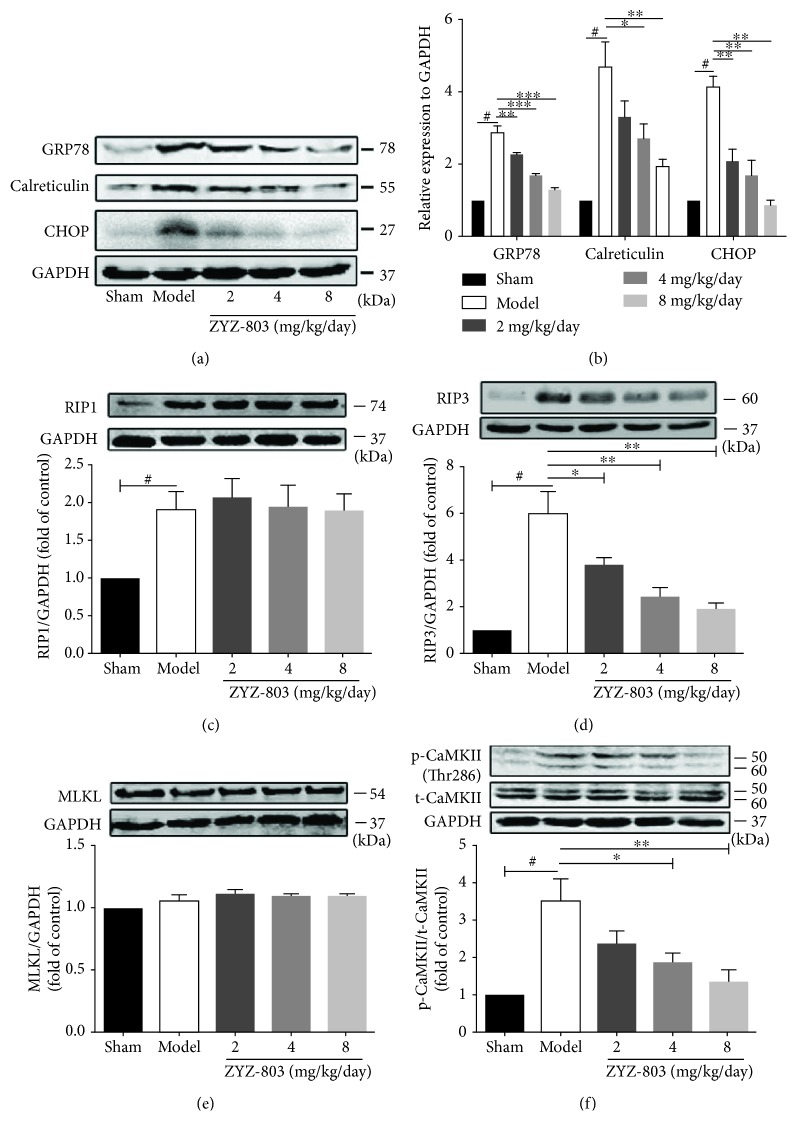
ZYZ-803 protects the heart from AMI injury by downregulating abnormal ERS and cardiac necroptosis. (a) Representative pictures of aberrant ERS-related protein expressions. (b) Analytical data of protein GRP78, calreticulin, and CHOP evaluated by Western blotting (*n* = 4 independent experiments). (c–f) Data of protein RIP1, RIP3, CaMKII, and MLKL expression (*n* = 4 independent experiments). Data are defined as mean ± SEM, one-way ANOVA. ^#^ *P* < 0.01 compared to the sham group; in comparison to the model group, ^∗^ *P* < 0.05, ^∗∗^ *P* < 0.01, and ^∗∗∗^ *P* < 0.001.

As stated above, both aberrant ERS and necroptosis are involved in the pathological process of AMI. To clarify whether ZYZ-803 could reduce ERS and necroptosis during AMI, we evaluated the change of related protein markers in mouse heart tissues using Western blotting. Results in Figures 4(a) and 4(b) illustrated a significant increase in the expression of GRP78, calreticulin, and CHOP in AMI mice compared to the sham group (^#^ *P* < 0.01), which was blocked significantly by ZYZ-803 preconditioning (^∗^ *P* < 0.05, ^∗∗^ *P* < 0.01, and ^∗∗∗^ *P* < 0.001). These results manifested amelioration of ZYZ-803 to aberrant ERS in AMI. Meanwhile, protein markers of necroptosis including RIP1, RIP3, MLKL, and phospho-CaMKII were measured in each group. The data showed that RIP1, RIP3, and phospho-CaMKII except MLKL were upregulated in the model mice (Figures 4(c)–4(f); ^#^ *P* < 0.001), while only RIP3 and phospho-CaMKII were apparently decreased by ZYZ-803 pretreatment (Figures 4(c)–4(f); ^∗^ *P* < 0.05, ^∗∗^ *P* < 0.001). These data revealed occurrence of aberrant ERS and necroptosis during AMI which were both blocked by ZYZ-803 preconditioning and also implied that cardiac necroptosis may be a downstream disastrous result of aberrant ERS during AMI.

### 3.5. ZYZ-803 Alleviates Necroptosis Induced by Abnormal ERS in Primary Cardiomyocytes

**Figure 5:**
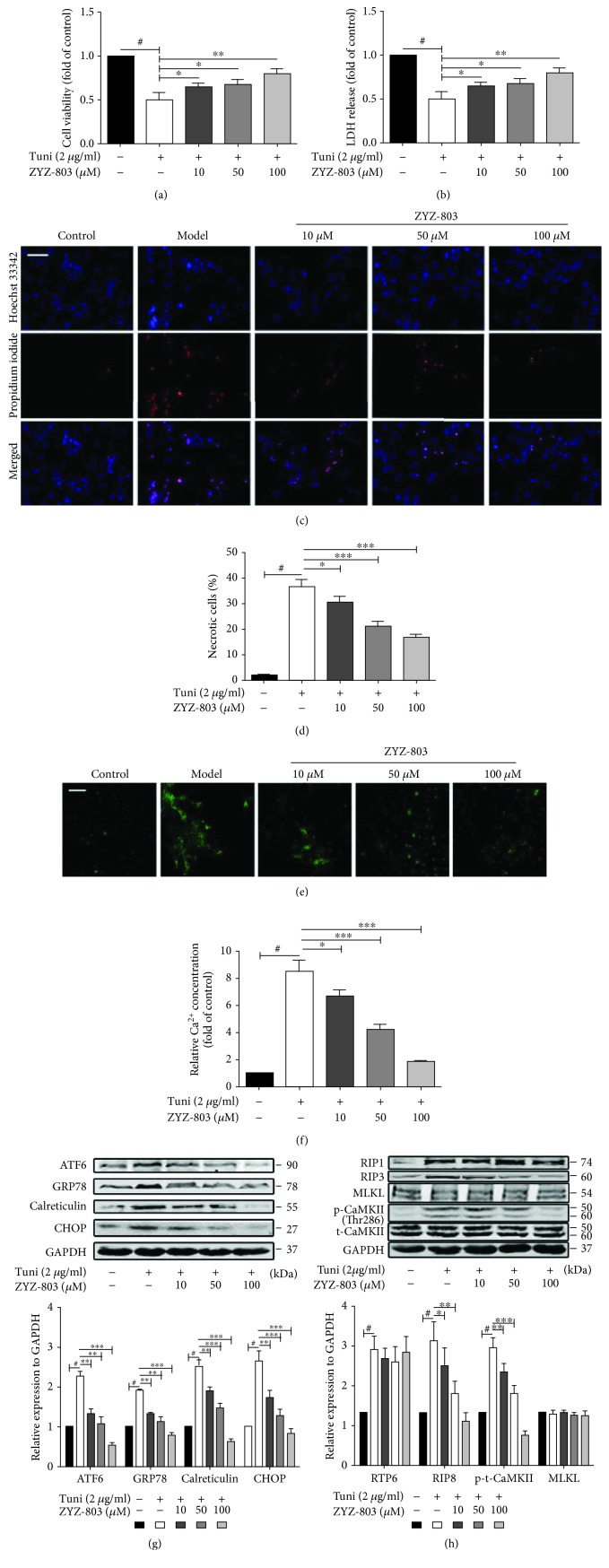
Cardiomyocyte necroptosis induced by abnormal ERS is alleviated by 1-hour pretreatment of ZYZ-803 *in vitro*. ERS was induced by tunicamycin 2 *μ*g/ml for 48 hours. (a) Influence of ZYZ-803 on cell viability by the MTT assay. (b) Averaged data of LDH release from cardiomyocytes. (c) Representative photographs of cell necrosis assayed by Hoechst 33342 and propidium iodide staining; scale bar (white): 50 *μ*m. (d) Data analysis of necrotic positive cells. (e) Intracellular [Ca^2+^]_i_ concentration visualized by confocal microscopy using the fluo-3/AM probe, a fluorescent Ca^2+^-indicator dye; scale bars (white): 50 *μ*m. (f) Statistic data of fluorescence intensity indicated intracellular calcium concentration. (g) Data of ERS-related protein expression. (h) Analysis of the expression of necroptosis protein markers. Data are defined as mean ± SEM, one-way ANOVA. ^#^ *P* < 0.01 compared to the control group; in comparison to the model group, ^∗^ *P* < 0.05, ^∗∗^ *P* < 0.01, and ^∗∗∗^ *P* < 0.001. *n* = 4 independent experiments.

To further verify the speculation that abnormal ERS may be an upstream inducer for myocardial necroptosis and that ZYZ-803 may play a similar role to this speculated ERS-related necroptosis just like in animals, an in vitro injury model of abnormal ERS induced by a typical ERS agonist tunicamycin (Tuni) was employed. 2 *μ*g/ml Tuni was chosen to treat primary neonatal ventricular cardiomyocytes (NRVCs) for 48 hours, and cell death was observably induced (Figure S2). The results, as shown in Figures 5(a) and 5(b), revealed that cellular necrosis indirectly indicated by cell viability and LDH release was obviously increased after Tuni treatment (^#^ *P* < 0.001), which was ameliorated by pretreatment of ZYZ-803 (^∗^ *P* < 0.05, ^∗∗^ *P* < 0.01). Likewise, after ZYZ-803 pretreatment, an obviously decreased level of necrosis (Figures 5(c) and 5(d); ^∗^ *P* < 0.05, ^∗∗∗^ *P* < 0.001) and intracellular Ca^2+^ (Figures 5(e) and 5(f); ^∗^ *P* < 0.05, ^∗∗∗^ *P* < 0.001) were observed by means of a fluorescent probe when compared to the Tuni group. In addition, upregulated expression of ERS-related proteins including ATF6, GRP78, calreticulin, and CHOP induced by Tuni was blocked by ZYZ-803 (Figure 5(g); ^#^ *P* < 0.01, ^∗∗^ *P* < 0.01, and ^∗∗∗^ *P* < 0.001). Furthermore, corresponding with the changes of ERS protein expression, preadministration of ZYZ-803 significantly blocked the upregulated level of RIP3 and phospho-CaMKII except RIP1 and MLKL (Figure 5(h); ^∗^ *P* < 0.05, ^∗∗^ *P* < 0.01, and ^∗∗∗^ *P* < 0.001), suggesting that downregulation of ZYZ-803 to ERS-related necroptosis in cardiomyocytes might be beyond depending on RIP1 and MLKL. These data demonstrated that ZYZ-803 could reduce cardiomyocyte necroptosis induced by abnormal ERS, and this is dependent on the RIP3-CaMKII signaling pathway.

### 3.6. ZYZ-803 Downregulates the RIP3-CaMKII Pathway to Reduce ERS-Related Necroptosis

**Figure 6:**
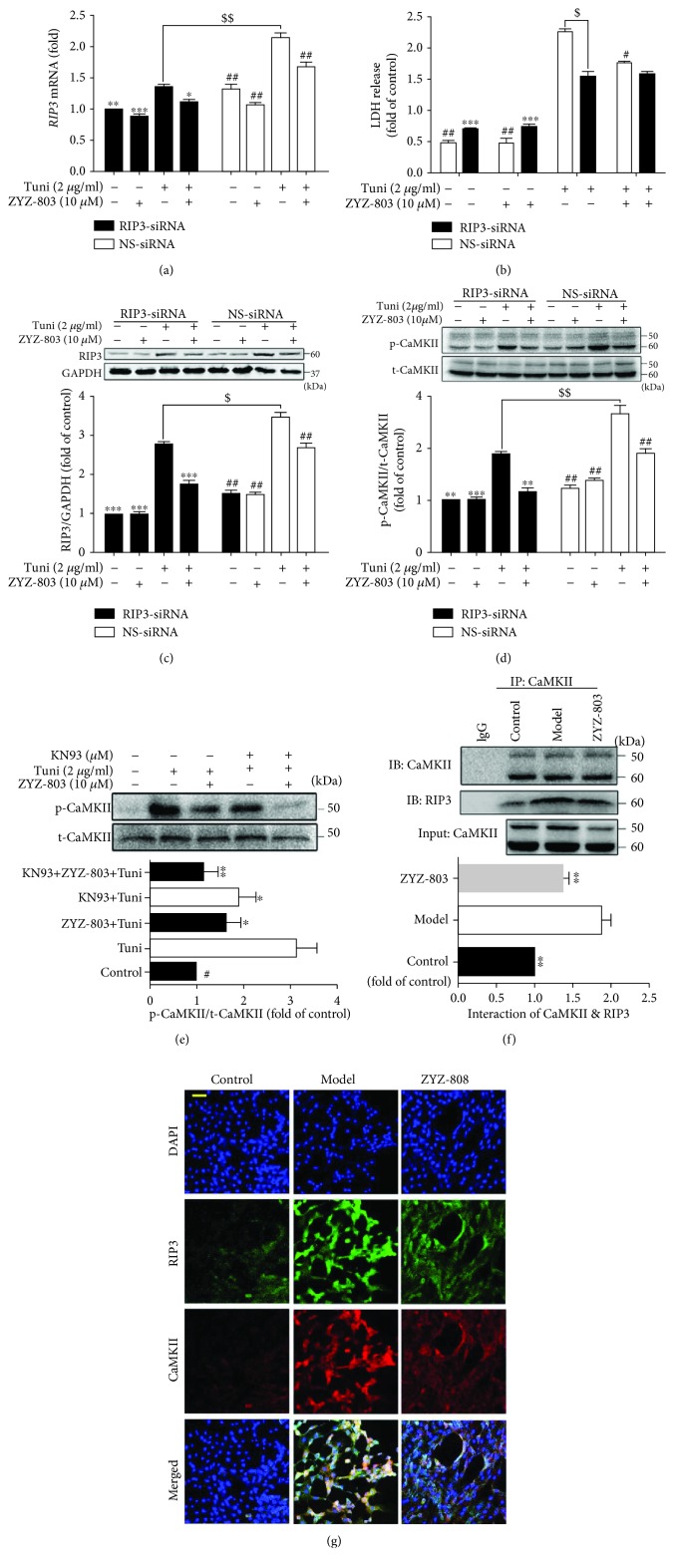
ZYZ-803 downregulates RIP3-CaMKII signaling in ERS injury. (a) Data of *RIP3* expression in cardiomyocytes treated by RIP3-siRNA/NS-siRNA. (b) LDH release of RIP3 knockdown and WT cardiomyocytes pretreated by ZYZ-803 (10 *μ*M) for 1 h before tunicamycin injury. (c) Protein expression of RIP3 in cardiomyocytes (*n* = 3 independent experiments). (d, e) Analytical data of p-/t-CaMKII expression in cardiomyocytes (*n* = 3 independent experiments). (f) Coimmunoprecipitation of RIP3 and CaMKII was blocked by ZYZ-803 preconditioning in heart tissues (*n* = 3 independent experiments). Data are defined as mean ± SEM, one-way ANOVA. Compared with the Tuni group (NS-siRNA), ^#^ *P* < 0.001, ^##^ *P* < 0.001; compared to the Tuni group (RIP3-siRNA), ^∗^ *P* < 0.05, ^∗∗^ *P* < 0.01, and ^∗∗∗^ *P* < 0.001; Tuni (NS-siRNA) versus the Tuni group (RIP3-siRNA), ^$^ *P* < 0.01, ^$$^ *P* < 0.05. (g) Immunofluorescent colocalization was decreased by ZYZ-803 in cardiomyocytes; scale bar (yellow): 50 *μ*m.

To further explore whether ZYZ-803 reduces cardiac necroptosis through downregulating RIP3-CaMKII signaling, we used small RNA interference and protein inhibitor to verify the effect of ZYZ-803 to ERS-induced necroptosis. As the results illustrated, transfection of RIP3-siRNA resulted in remarkable block of upregulated mRNA and protein expression induced by Tuni, which was similar to the effect of ZYZ-803 pretreatment without *RIP3* knockdown (Figures 6(a) and 6(b); ^$^ *P* < 0.01, ^$$^ *P* < 0.001, ^∗^ *P* < 0.05, and ^∗∗∗^ *P* < 0.001). Furthermore, after RIP3-siRNA transfection, paralleled results were observed in the downstream of RIP3 including LDH release and CaMKII activity (Figures 6(c) and 6(d); ^$^ *P* < 0.01, ^$$^ *P* < 0.001, ^#^ *P* < 0.05, ^##^ *P* < 0.01, and ^∗∗∗^ *P* < 0.001). Meanwhile, downregulation of phospho-CaMKII by ZYZ-803 was similar to the CaMKII inhibitor KN93, and cotreatment of ZYZ-803 and KN93 could further decrease phospho-CaMKII (Figure 6(e); ^∗^ *P* < 0.05, ^∗∗^ *P* < 0.01). These results suggested that ZYZ-803 could downregulate the RIP3-CaMKII signaling pathway to reduce cardiomyocyte necroptosis.

**Figure 7:**
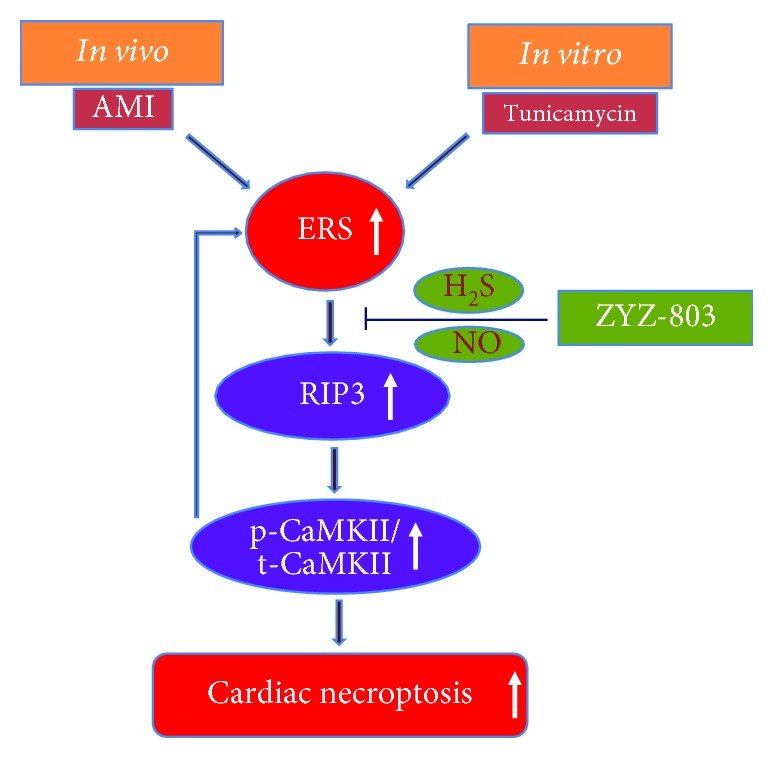
Schematic diagram for the new mechanism of ZYZ-803 in ER stress-related cardiac necroptosis. Aberrant ERS could be evoked during AMI and by its typical agonist tunicamycin in cardiomyocytes, which potentially induces myocardial necroptosis through the RIP3-CaMKII pathway. ZYZ-803, a dual donor for gasotransmitters H_2_S and NO, could downregulate this ERS-related necroptosis at upstream of RIP3-CaMKII signaling through H_2_S and NO balance and finally protects the heart from acute myocardial ischemia injury.

Based on the above point that ZYZ-803 could downregulate RIP3-CaMKII pathway, we next tried to detect the effect of ZYZ-803 to the interaction of RIP3 and CaMKII. In cardiomyocytes, as shown in the immunofluorescent graphs in Figure 6(g), colocalization of RIP3 and CaMKII by double immunofluorescent staining in the ZYZ-803 group was apparently reduced compared to the model one. Additionally, formation of an immunoprecipitated complex between RIP3 and CaMKII in AMI heart tissues was significantly decreased in the ZYZ-803 preintervention group (Figure 6(f); ^∗∗^ *P* < 0.01, ^∗∗∗^ *P* < 0.001). These data further verified the regulation of ZYZ-803 to the RIP3-CaMKII signaling pathway as the paradigm shows in Figure 7.

## 4. Discussion

AMI is largely caused by epicardial coronary artery blockage from vulnerable plaques. This supports the rationale of using traditional approaches like fibrinolytic and antithrombotic therapies as well as percutaneous and surgical revascularization approaches to eliminate these obstructions. Some newly developed strategies, as described herein, focus on cardioprotection. This strategy exploits the use of drug entities that protect the heart from stress-induced injury, to prevent cell necrosis and to promote recovery. Cardiac necroptosis plays a pivotal pathological role in myocardial ischemic injury and has been seen as a new therapeutic target for ischemic heart injury [37, 38]. Meanwhile, H_2_S and NO are recognized as gasotransmitters with widely pathophysiological roles in cardiovascular diseases, and both of them are dysregulated in the pathogenesis of myocardial ischemic injury, which can be rescued by H_2_S and/or NO donors through different mechanisms including complex interplays of H_2_S and NO [30, 39, 40]. However, there are no reports about the role of H_2_S or NO in cardiac necroptosis during myocardial ischemia. From this perspective, in view of the proangiogenic role of ZYZ-803 by cooperative effect of H_2_S and NO in heart failure [32], we inspiredly evaluated the cardioprotective potential of ZYZ-803 pretreatment after myocardial injury by targeting cardiac necroptosis *in vivo* and *in vitro*. The current results from animal experiments indeed revealed that ZYZ-803 pretreatment could block cardiac function damage and histopathologic deterioration in a dose-dependent manner (Figures 1 and 2 and Table S1), and cardiac necroptosis was also apparently decreased by ZYZ-803 (Figures 4(c)–4(f)). In parallel, cardiomyocyte necrosis induced by oxygen-glucose deprivation (OGD) and tunicamycin was significantly alleviated by ZYZ-803 pretreatment (Figure S3(a), and Figures 5(a)–5(f)). Thus, these results firstly provide evidences for the cardioprotection of ZYZ-803 pretreatment through prevention of cardiac necroptosis during acute myocardial ischemia injury.

It has been widely recognized that the disturbance of endogenous production of gasotransmitters NO and H_2_S is associated with the pathology of AMI [41, 42]. Thus, it is theoretically applicable to generate protective effect on AMI injury through rescuing the dysregulation of these two small gasotransmitters. Previous studies from our lab have revealed that exogenous H_2_S donors such as sodium hydrosulfide and S-propargyl-cysteine can reverse dysregulation in H_2_S homeostasis following AMI injury through different mechanisms [33, 42–45], and the deteriorative change after myocardial infarction is prevented by various NO donors [46–48]. In fact, it has been reported that synchronous administration of NO and H_2_S donors could generate additive protective effect in ischemic heart injury [48]. In our research, we are the first to explore a novel combined donor of H_2_S and NO, namely, ZYZ-803, in acute myocardial ischemia. Results showed that decreased levels of H_2_S and NO in serum and heart tissues following AMI are reversed by ZYZ-803 pretreatment (Figures 3(a)–3(d)). Moreover, ZYZ-803 could reverse the aberrant expressions of H_2_S synthase CBS and CSE in AMI in addition to increasing the activity of eNOS (Figures 3(c)–3(f)). Of note, in our results, we detected upregulated expression of CSE but a low H_2_S level after AMI, which is contrary to the previous study [45]. The different time points of measurement of CSE and the H_2_S level in the current study could be one of the reasons. Additionally, because CSE is sensitive to the change of the H_2_S level, we speculate that it may be a transiently compensatory mechanism of CSE expression in risk cardiomyocytes to respond to the sharply decreased H_2_S after acute myocardial ischemia. Considering that other H_2_S synthetases, CBS, for example, can also contribute to H_2_S generation, though not mainly as CSE in the cardiovascular system, we detected the change of CBS expression and found that CBS was decreased after AMI, which was blocked by ZYZ-803. These results just demonstrated an imbalanced change of H_2_S synthetase systems that could be revised by ZYZ-803. On the other hand, it is very interesting that ZYZ-803 could also influence the synthases of H_2_S and NO besides its direct release. We believe that there must be some unknown and complicated regulation mechanisms between H_2_S and NO molecules and their synthases. However, more daringly, we speculate that partial H_2_S and NO molecules released from ZYZ-803 might be a positive signal to stimulate their synthases for more H_2_S and NO productions. By and large, from these results, we conclude that ZYZ-803 may play its protective role through cooperatively regulating the homeostases of H_2_S and NO during AMI injury.

As mentioned, abnormal ERS is involved in the pathogenesis of AMI, and cardiac necroptosis has been revealed as a pivotal event in myocardial ischemia. In addition, in L929 cells, it has been shown that sustained ERS can result in necroptosis [26]. Thus, sustained ERS and necroptosis likely occur concurrently, and necroptosis might be a secondary outcome of sustained ERS during acute myocardial ischemia. In fact, we reported on the occurrence of sustained ERS and necroptosis in AMI mice and OGD cardiomyocytes, and the results clearly showed that ZYZ-803 preconditioning downregulated the expression of markers linked to these processes (Figure 4, Figure S3(b)). Furthermore, in order to verify the speculation that abnormal ERS may also induce necroptosis in cardiomyocytes, tunicamycin, a typical agonist for sustained ERS, was used to treat NRVCs, and indeed, necroptosis was remarkably induced besides sustained ERS, which was detected by decreased cell viability and increased LDH release, positive necrotic cells, intracellular Ca^2+^ overload, and representative protein markers. However, this necroptosis induced by sustained ERS was alleviated by pretreated ZYZ-803 (Figure 5). Therefore, these results implied that ZYZ-803 could ameliorate necroptosis secondary to ERS in the condition of acute myocardial ischemia.

It is recognized that the classical signaling pathway of necroptosis is dependent on the RIP1-RIP3-MLKL axis, namely, “RIP1-dependent” signaling. Typically, this pathway is usually induced by TNF-*α* and involves caspase-8 and the death receptor TNFR1 [37, 49]. At this context, uncleaved RIP1 and activated RIP3 form a complex called the “necrosome,” which further recruits MLKL, a pseudokinase, to execute necroptosis [50, 51]. In L929 cells, sustained ERS promotes necroptosis via the RIP1-RIP3-MLKL signaling pathway [26]. However, in our work, even though obvious overexpression of RIP1 and RIP3 appeared, upregulated CaMKII activity rather than change of MLKL expression was observed both in AMI mice and in tunicamycin-induced NRVCs (Figures 4(c)–4(h) and Figure 5(h)). The possible explanation for this difference may be that MLKL does not participate in the regulation of cardiac necroptosis induced by aberrant ERS just as its negligible role in cardiac necroptosis was triggered by ischemic-reperfusion and oxidative stress [11]. Furthermore, it is interesting to note that only RIP3 and phospho-CaMKII were downregulated by ZYZ-803 preconditioning in the condition of our research (Figures 4 and 5). At this point, these results suggest that ZYZ-803 mediates its effects on ERS-related necroptosis in myocardium through the RIP3-CaMKII pathway rather than the classical “RIP1-RIP3-MLKL” axis.

Finally, in terms of the mechanisms of action for ZYZ-803, we believe that the alleviation of ERS-mediated necroptosis by ZYZ-803 is dependent on the downregulation of RIP3-CaMKII signaling in cardiomyocytes. Previous studies have shown that RIP3 knockout decreases myocardial necroptosis in myocardial infarction [9]. Similarly, in our study, RIP3 knockdown by siRNA blocked RIP3 overexpression and CaMKII phosphorylation following ERS-related necroptosis with decreased LDH release, similar to the effects seen in the ZYZ-803 pretreatment groups (Figures 6(a)–6(d)). However, ZYZ-803 pretreatment failed to further downregulate CaMKII phosphorylation after RIP3 knockdown, again suggesting an upstream effect of ZYZ-803 on RIP3. Furthermore, downregulation of ZYZ-803 to phospho-CaMKII was similar to that of the CaMKII inhibitor KN93 (Figure 6(e)). Interestingly, cotreatment of ZYZ-803 and KN93 had an additive inhibition to CaMKII (Figure 6(e)). Because *S*-sulfhydration and *S*-nitrosylation of CaMKII, respectively, by H_2_S and NO could inhibit activity of CaMKII [52, 53], we speculate that as a dual donor for H_2_S and NO, ZYZ-803 might *S*-sulfhydrate and *S*-nitrosylate CaMKII to indirectly reduce phospho-CaMKII, which is beneficial to block cardiac necroptosis. From these results, we demonstrated that alleviation of ERS-related cardiac necroptosis by ZYZ-803 pretreatment is mediated by downregulating RIP3-CaMKII signaling. So, we finally were in turn to verify that ZYZ-803 could downregulate the interaction of RIP3 and CaMKII as determined using fluorescent colocalization in cells and immunoprecipitate blotting in animal tissues (Figures 6(e) and 6(f)). However, we have yet to determine the cellular target of ZYZ-803 that occurs upstream of RIP3. As such, further experiments will determine the mechanism of ERS-related necroptosis in myocardial injury in order to reveal the effect and targets of H_2_S and NO in our model.

In conclusion, we herein have been the first to reveal that (1) the RIP3-CaMKII axis is mainly involved in ERS-related necroptosis in AMI; (2) the cardioprotective role of ZYZ-803 in AMI is realized by its regulation to aberrant H_2_S and NO homeostases via releasing both H_2_S and NO; (3) ZYZ-803 alleviates ERS-related necroptosis during AMI through downregulating necroptosis signaling RIP3-CaMKII. In closing, we report that ZYZ-803 protects heart tissues against acute myocardial ischemia via the targeting of ERS-related necroptosis. This work supports future studies in the development of ZYZ-803 as a clinically significant candidate for use in the treatment of AMI.

## Data Availability

The data used to support the findings of this study are included in the article and the supplementary information file. And the datasets are available from the corresponding authors on reasonable request.

## Abbreviations

AAR: Area at risk
AMI: Acute myocardial infarction
ATF6: Activating transcription factor 6
CaMKII: Ca^2+^-calmodulin-dependent protein kinase
CBS: Cystathionine *β*-synthase
CHOP: CCAAT/enhancer-binding protein homologous protein
CSE: Cystathionine-*γ*-lyase
DMEM: Complete Dulbecco's modified Eagle medium
eNOS: Endothelial NO synthase
ER: Endoplasmic reticulum
ERS: Endoplasmic reticulum stress
GRP78: Glucose-related protein 78
HBSS: Hank's Balanced Salt Solution
HE: Hematoxylin and eosin
HRP: Horseradish peroxidase
IP: Immunoprecipitation
IS: Infarct size
LDH: Lactic dehydrogenase
LVEF: Left ventricular ejection fraction
LVFS: Left ventricular fractional shortening
LVSD: Left ventricular systolic diameter
LVSV: Left ventricular systolic volume
MI: Myocardial infarction
MLKL: Mixed lineage kinase domain-like protein
NRVCs: Neonatal rat ventricular cardiomyocytes
Phospho-CaMKII: Phosphorylated CaMKII
RIP1/3: Receptor-interacting protein 1/3
TTC: Triphenyltetrazolium chloride
Tuni: Tunicamycin.

## Supplementary Materials

Supplementary Materials Table S1: data of parameters indicating cardiac function in each group especially supplemented with the sham+ZYZ-803 group, which showed no side effect of ZYZ-803 preconditioning to the normal heart. Figure S1: cytotoxicity test of ZYZ-803 to cardiomyocytes showed that the ZYZ-803 dosage of more than 200 *μ*M emerged cardiac injury. Primary cardiomyocytes were treated with ZYZ-803 for 1 hour. Data are defined as mean ± SEM, one-way ANOVA. Compared to group 0 *μ*M, ^∗^ *P* < 0.05, ^∗∗∗^ *P* < 0.001. *n* = 3 independent experiments. Figure S2: induction of cardiomyocyte death by tunicamycin (Tuni) treatment showed that Tuni treatment at 2 *μ*g/ml for 48 h could remarkably induce cardiomyocyte death. Data are defined as mean ± SEM, one-way ANOVA. Compared to group 0 *μ*g/ml, ^∗^ *P* < 0.05, ^∗∗^ *P* < 0.01, and ^∗∗∗^ *P* < 0.001. *n* = 3 independent experiments. Figure S3: protection of ZYZ-803 against myocardial injury induced by oxygen-glucose deprivation (OGD). Primary cardiomyocytes with or without 1 h ZYZ-803 preadministration were treated with glucose-free medium and incubated in 95% N_2_ and 5% CO_2_ for 16 hours. (a) Assay of LDH release revealed reduction of ZYZ-803 to cardiomyocyte death induced by OGD. (b) Immunoblotting of protein expressions for ERS and necroptosis illustrated downregulation of ZYZ-803 to ERS and necroptosis induced by OGD. Data are defined as mean ± SEM, one-way ANOVA. Compared to the OGD group, ^#^ *P* < 0.01, ^∗^ *P* < 0.05, and ^∗∗^ *P* < 0.01. *n* = 3 independent experiments. Figure S4: no significant change of the heart rate in each experimental group. Figure S5: effect of ZYZ-803 to apoptosis. Primary cardiomyocytes with or without 1 h ZYZ-803 preadministration were treated with 2 *μ*g/ml Tuni for 48 hours, and then, apoptosis protein markers were detected by Western blotting. Data are defined as mean ± SEM, one-way ANOVA. Compared to the model group, ^∗^ *P* < 0.05, ^∗∗^ *P* < 0.01, and ^∗∗∗^ *P* < 0.001. *n* = 3 independent experiments. Figure S6: influence of ZYZ-803 to RIP3 and CaMKII detected by immunohistochemistry. Scale bar (black): 100 *μ*m.
