# Supplementary material for: ZYZ-803 Mitigates Endoplasmic Reticulum Stress-Related Necroptosis after Acute Myocardial Infarction through Downregulating the RIP3-CaMKII Signaling Pathway
Source: Oxid Med Cell Longev. 2019 Jun 2;2019:6173685. doi: 10.1155/2019/6173685 (PMC6589311; doi:10.1155/2019/6173685)
Supplement: Supplementary Materials — Table S1: data of parameters indicating cardiac function in each group especially supplemented with the sham+ZYZ-803 group, which showed no side effect of ZYZ-803 preconditioning to the normal heart. Figure S1: cytotoxicity test of ZYZ-803 to cardiomyocytes showed that the ZYZ-803 dosage of more than 200 μM emerged cardiac injury. Primary cardiomyocytes were treated with ZYZ-803 for 1 hour. Data are defined as mean ± SEM, one-way ANOVA. Compared to group 0 μM, ∗ P < 0.05, ∗∗∗ P < 0.001. n = 3 independent experiments. Figure S2: induction of cardiomyocyte death by tunicamycin (Tuni) treatment showed that Tuni treatment at 2 μg/ml for 48 h could remarkably induce cardiomyocyte death. Data are defined as mean ± SEM, one-way ANOVA. Compared to group 0 μg/ml, ∗ P < 0.05, ∗∗ P < 0.01, and ∗∗∗ P < 0.001. n = 3 independent experiments. Figure S3: protection of ZYZ-803 against myocardial injury induced by oxygen-glucose deprivation (OGD). Primary cardiomyocytes with or without 1 h ZYZ-803 preadministration were treated with glucose-free medium and incubated in 95% N2 and 5% CO2 for 16 hours. (a) Assay of LDH release revealed reduction of ZYZ-803 to cardiomyocyte death induced by OGD. (b) Immunoblotting of protein expressions for ERS and necroptosis illustrated downregulation of ZYZ-803 to ERS and necroptosis induced by OGD. Data are defined as mean ± SEM, one-way ANOVA. Compared to the OGD group, # P < 0.01, ∗ P < 0.05, and ∗∗ P < 0.01. n = 3 independent experiments. Figure S4: no significant change of the heart rate in each experimental group. Figure S5: effect of ZYZ-803 to apoptosis. Primary cardiomyocytes with or without 1 h ZYZ-803 preadministration were treated with 2 μg/ml Tuni for 48 hours, and then, apoptosis protein markers were detected by Western blotting. Data are defined as mean ± SEM, one-way ANOVA. Compared to the model group, ∗ P < 0.05, ∗∗ P < 0.01, and ∗∗∗ P < 0.001. n = 3 independent experiments. Figure S6: influence of ZYZ-803 to RIP3 and CaMKII detected [file 6173685.f1.pdf]

Supplementary materials

TABLE S1. Data of parameters indicating cardiac function in each group especially supplemented with the sham+ZYZ-803 group, which showed no side effect of ZYZ-803 preconditioning to normal heart. FIGURE S1. Cytotoxicity test of ZYZ-803 to cardiomyocytes showed ZYZ-803 dosage more than 200μM emerged cardiac injury. Primary cardiomyocytes were treated with ZYZ-803 for 1 hour. FIGURE S2. Induction of cardiomyocyte death by tunicamycin (Tuni) treatment showed that Tuni treatment at 2μg/ml for 48h could remarkably induce cardiomyocyte death. FIGURE S3. Protection of ZYZ-803 against myocardial injury induced by oxygen-glucose deprivation (OGD). Primary cardiomyocytes with or without 1h ZYZ-803 pre-administration were treated with glucose-free medium and incubated in 95% N_2 and 5% CO_2 for 16 hours. (a) Assay of LDH release revealed reduce of ZYZ-803 to cardiomyocyte death induced by OGD. (b) Immunoblotting of protein expressions for ERS and necroptosis illustrated downregulation of ZYZ-803 to ERS and necroptosis induced by OGD. FIGURE S4. No significant change of heart rate in each experimental group after AMI and ZYZ-803 pretreatment. FIGURE S5. ZYZ-803 could downregulate cardiomyocyte apoptosis induced by Tuni. FIGURE S6. Influence of ZYZ-803 to RIP3 and CaMKII detected by immunohistochemistry.

TABLE S1: Data of parameters indicating cardiac function in each group especially supplemented with the sham+ZYZ-803 group, which showed no side effect of ZYZ-803 preconditioning to normal heart

Measurement	LVEF(%)	LVFS(%)	LVSD(mm)	LVSV(ul)
**Sham**	70.55±7.84	39.64±5.99	2.24±0.38	17.72±7.70
**Sham+ZYZ-803(8mg/kg/day)**	66.63±4.47	36.29±3.23	2.43±0.36	21.41±8.26
**Model**	19.14±4.62	8.64±2.17	4.17±0.59	79.20±26.54
**Model+ZYZ-803(2mg/kg/day)**	21.94±8.48	10.08±4.23	4.11±0.41	75.35±17.86
**Model+ZYZ-803(4mg/kg/day)**	30.43±10.14	14.32±5.07	3.82±0.78	66.08±31.58
**Model+ZYZ-803(8mg/kg/day)**	44.55±7.22	22.62±2.56	3.09±0.41	41.07±13.85

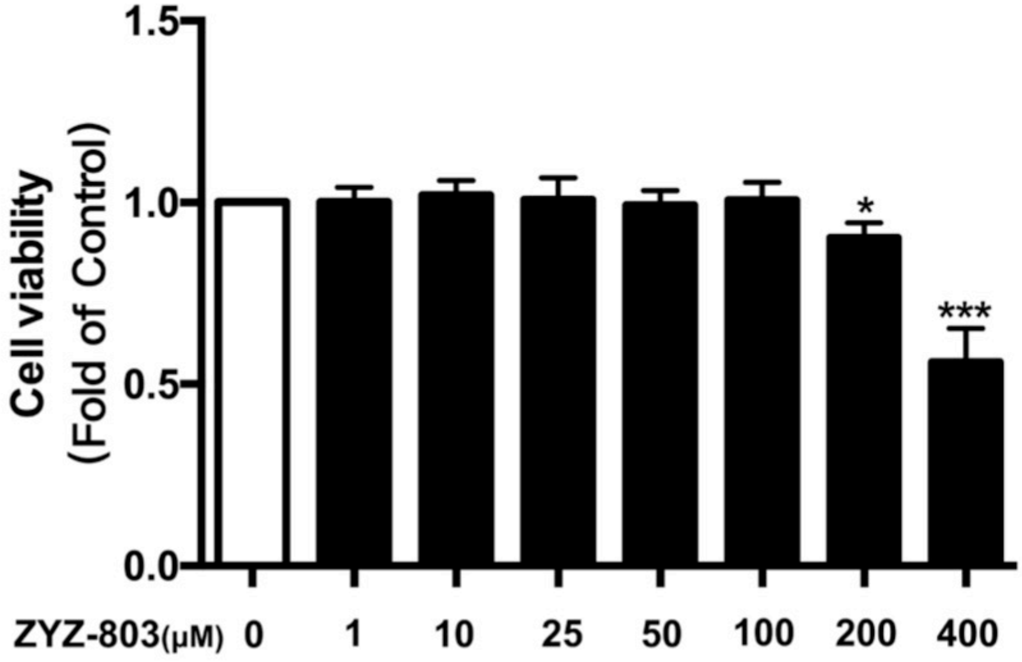

FIGURE S1: Cytotoxicity test of ZYZ-803 to cardiomyocytes showed ZYZ-803 dosage more than 200μM emerged cardiac injury. Primary cardiomyocytes were treated with ZYZ-803 for 1 hour. Data are defined as mean ± SEM, one-way ANOVA. Compared to group 0 μM, *P<0.05, ***P<0.001. n=3 independent experiments.

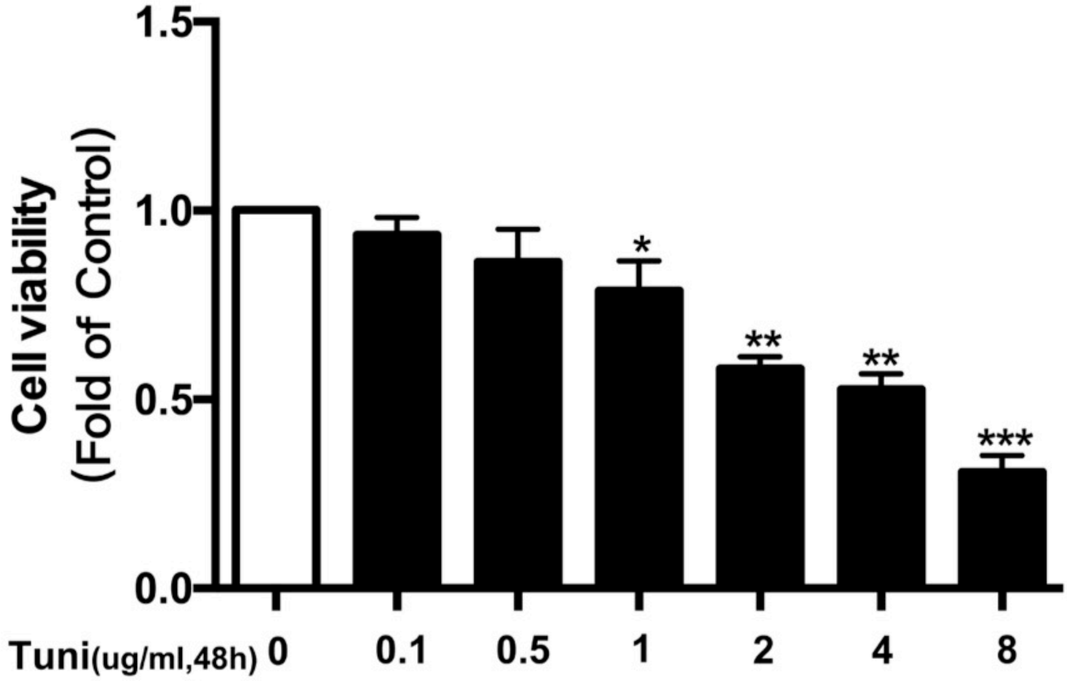

FIGURE S2: Induction of cardiomyocyte death by tunicamycin (Tuni) treatment showed that Tuni treatment at 2μg/ml for 48h could remarkably induce cardiomyocyte death. Data are defined as mean ± SEM, one-way ANOVA. Compared to group 0 ug/ml, *P<0.05, **P<0.01, ***P<0.001. n=3 independent experiments.

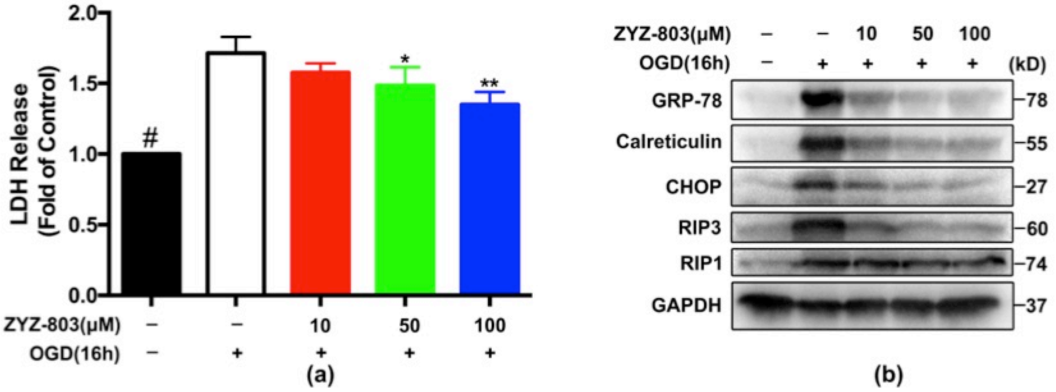

FIGURE S3: protection of ZYZ-803 against myocardial injury induced by oxygen-glucose deprivation (OGD). Primary cardiomyocytes with or without 1h ZYZ-803 pre-administration were treated with glucose-free medium and incubated in 95% N_2 and 5% CO_2 for 16 hours. (a) Assay of LDH release revealed reduce of ZYZ-803 to cardiomyocyte death induced by OGD. (b) Immunoblotting of protein expressions for ERS and necroptosis illustrated downregulation of ZYZ-803 to ERS and necroptosis

induced by OGD. Data are defined as mean ± SEM, one-way ANOVA. Compared to OGD group, #$P<0.01$, *$P<0.05$, **$P<0.01$. n=3 independent experiments.

Heart Rate Change after AMI and treatment of ZYZ-803

	Sham	Model	ZYZ-803（mg/kg/day）		
			2	4	8
Mean	514.8	505.3	530.0	516.5	527.8
Std. Deviation	58.16	62.19	34.70	76.97	54.79

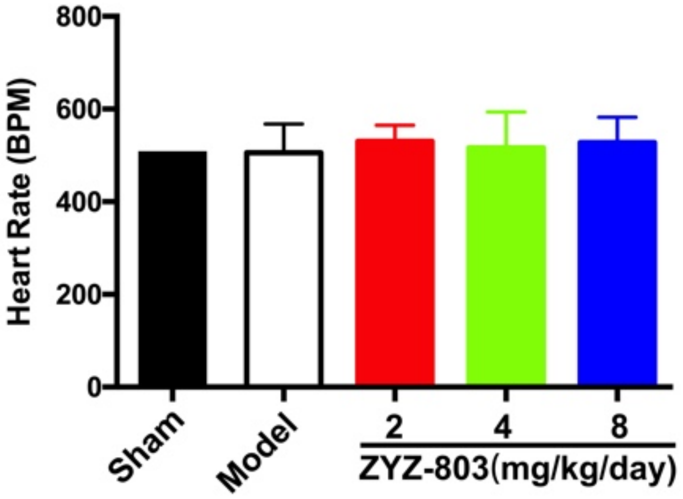

FIGURE S4: No significant change of heart rate in each experimental group.

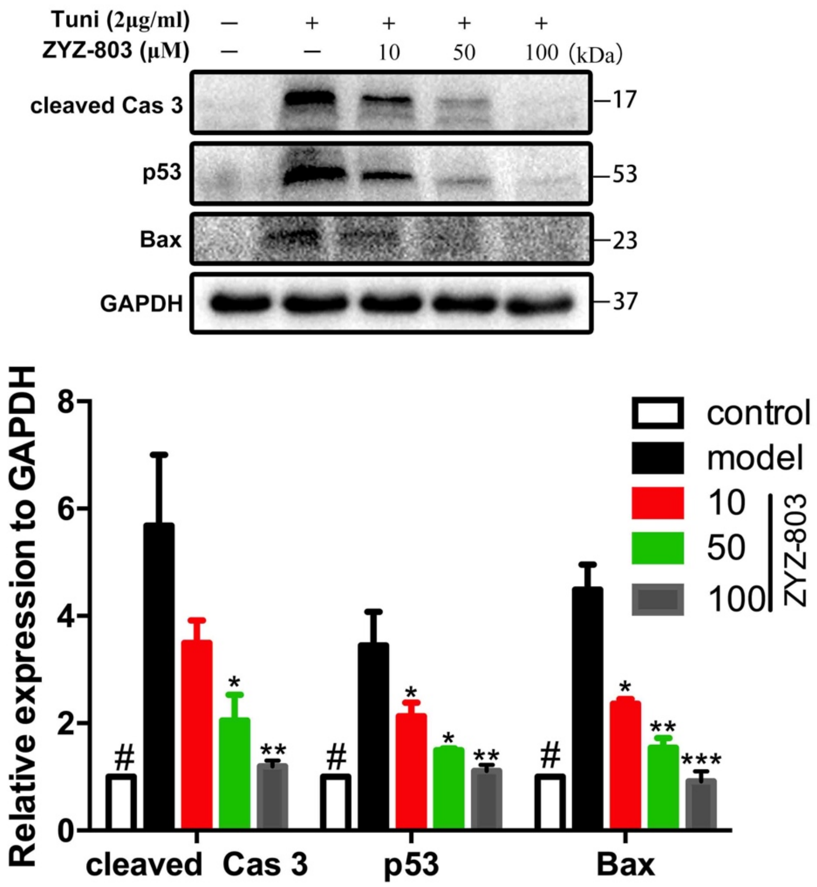

Figure S5. Effect of ZYZ-803 to apoptosis. Primary cardiomyocytes with or without 1h ZYZ-803 pre-administration were treated with 2μg/ml Tuni for 48 hours, and then apoptosis protein markers were detected by western blotting. Data are defined as mean ± SEM, one-way ANOVA. Compared to model group, **P*<0.05, ***P*<0.01, ****P*<0.001. n=3 independent experiments.

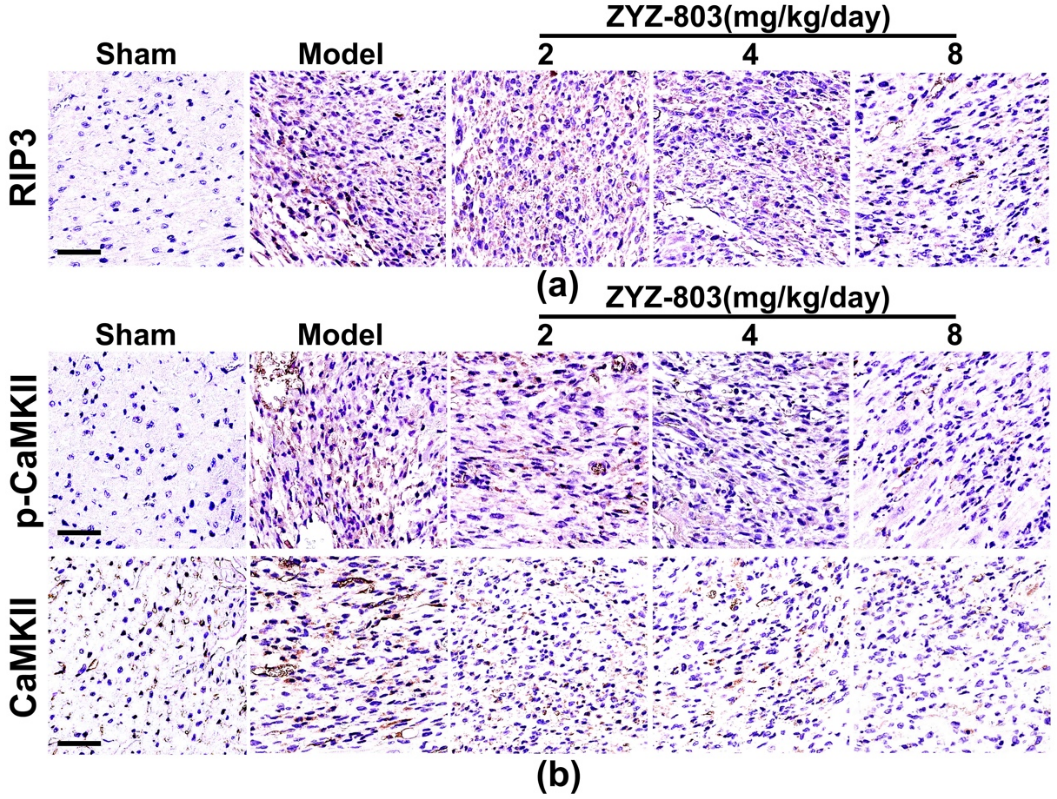

Figure S6. Influence of ZYZ-803 to RIP3 and CaMKII detected by immunohistochemistry. Scale bar (black), 100 μm.
